# TNFR1 and TNFR2 in the Control of the Life and Death Balance of Macrophages

**DOI:** 10.3389/fcell.2019.00091

**Published:** 2019-05-29

**Authors:** Harald Wajant, Daniela Siegmund

**Affiliations:** Division of Molecular Internal Medicine, Department of Internal Medicine II, University Hospital Würzburg, Würzburg, Germany

**Keywords:** apoptosis, necroptosis, TNF, TNFR1, TNFR2, ripk1, ripk3, caspase-8

## Abstract

Macrophages stand in the first line of defense against a variety of pathogens but are also involved in the maintenance of tissue homeostasis. To fulfill their functions macrophages sense a broad range of pathogen- and damage-associated molecular patterns (PAMPs/DAMPs) by plasma membrane and intracellular pattern recognition receptors (PRRs). Intriguingly, the overwhelming majority of PPRs trigger the production of the pleiotropic cytokine tumor necrosis factor-alpha (TNF). TNF affects almost any type of cell including macrophages themselves. TNF promotes the inflammatory activity of macrophages but also controls macrophage survival and death. TNF exerts its activities by stimulation of two different types of receptors, TNF receptor-1 (TNFR1) and TNFR2, which are both expressed by macrophages. The two TNF receptor types trigger distinct and common signaling pathways that can work in an interconnected manner. Based on a brief general description of major TNF receptor-associated signaling pathways, we focus in this review on research of recent years that revealed insights into the molecular mechanisms how the TNFR1-TNFR2 signaling network controls the life and death balance of macrophages. In particular, we discuss how the TNFR1-TNFR2 signaling network is integrated into PRR signaling.

## Introduction

Tumor necrosis factor-alpha (TNF) is a highly pleiotropic cytokine that affects practically any type of cell. It triggers cellular responses reaching from the induction of inflammatory gene expression programs, over the stimulation of cellular proliferation and differentiation to the activation of cellular suicide programs such as apoptosis and necroptosis (Wajant et al., 2003; Wajant and Scheurich, 2011; Brenner et al., 2015).

**FIGURE 1:**
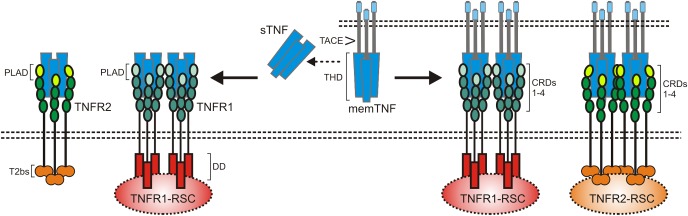
The TNF-TNFR1-TNFR2 system. As other receptors of the TNFRSF, TNFR1 and TNFR2 are characterized by cysteine-rich domains (CRD) in their extracellular part. TNFR1 harbors furthermore a death domain (DD) and TNFR2 a TRAF2 binding site (T2bs). TNF occurs in two forms, as a membrane-bound trimeric ligand (memTNF) and as a soluble likewise trimeric molecule (sTNF). TACE processes memTNF to sTNF. Please note, memTNF stimulates both TNF receptors while sTNF largely fails to stimulate TNFR2 despite high-affinity binding. PLAD, pre-ligand binding assembly domain.

Tumor necrosis factor-alpha is the name giving and prototypic ligand of the TNF superfamily (TNFSF). It is expressed (i) as a type II single spanning transmembrane protein and (ii) as a soluble variant which is released from the transmembrane form by proteolytic processing in the stalk region which separates the characteristic TNF homology domain (THD) from the transmembrane and the intracellular domain (Locksley et al., 2001; Bodmer et al., 2002; Figure 1). Since the THD mediates self-assembly into trimeric molecules and receptor binding, both the transmembrane and soluble form of TNF interact with the two know receptors of TNF, TNF receptor 1 (TNFR1), and TNFR2 (Figure 1). Both receptors of TNF are typical representatives of the TNF receptorsuperfamily (TNFRSF). As such, TNFR1 and TNFR2 are single-spanning type I transmembrane proteins characterized by having several cysteine-rich domains (CRDs) in their extracellular domain (Locksley et al., 2001; Bodmer et al., 2002). Soluble forms of TNFR1 and TNFR2 have also been described and result from alternative splicing or shedding (Philippe et al., 1993; Taylor, 1994; Galve-de Rochemonteix et al., 1996; Lainez et al., 2004; Gregory et al., 2012). The soluble TNF receptor variants inhibit TNF by competing with the cellular receptor species for TNF binding but possibly also by acting as dominant-negative molecules. Indeed, the N-terminal CRDs of TNFR1 and TNFR2 are not directly involved in ligand binding but mediate inactive self-association in the absence of ligand (Chan et al., 2000). This part of the TNF receptors has therefore been named pre-ligand binding assembly domain (PLAD) and seems to be a prerequisite for ligand binding and subsequent formation of active receptor complexes (Chan et al., 2000). Thus, soluble TNF receptor molecules might also act as TNF inhibitors by formation of inactive complexes with cellular TNF receptors by PLAD-PLAD interaction, but this issue has not been clarified yet.

TNFR1 and TNFR2 belong to different subgroups of the TNFRSF. TNFR1 is a death receptor (DR) and harbors a death domain (DD) in its cytoplasmic part (Tartaglia et al., 1993). The DD is a conserved type of protein-protein interaction domain which enables DRs to interact homotypically with cytoplasmic proteins also harboring a DD (Park et al., 2007). DD-containing signaling proteins link TNFR1 to cytotoxic signaling pathways triggering apoptosis or necroptosis but also allow engagement of signaling pathways that activate transcription factors of the nuclear factor of kappa B (NFκB) family or kinases of the MAP kinase family (Wajant et al., 2003; Wajant and Scheurich, 2011; Brenner et al., 2015). There exist several mechanisms, described below in more detail, that suppress cytotoxic signaling by TNFR1 so that proinflammtory, gene inductive signaling can be considered as the default mode of TNFR1 activity. TNFR2 has no DD and is a prototypic TNF receptor associated factor (TRAF)-interacting TNFRSF receptor (Xie, 2013). Thus, there is a short amino acid motif near the C-terminus of TNFR2 which enables recruitment of the adapter protein TRAF2 and TRAF2-associated proteins such as TRAF1 and cellular inhibitor of apoptosis protein 1 (cIAP1) and cIAP2 (Xie, 2013). TNFR2 has therefore no intrinsic cell death inducing activity but stimulates NFκB signaling and activation of various kinases (Wajant et al., 2003). The transmembrane and soluble form of TNF bind with high affinity to the two TNF receptor types and crystallographic data revealed a similar structural mode of ligand binding by TNFR1 and TNFR2 (Banner et al., 1993; Mukai et al., 2010). Nevertheless, there is a striking difference in the TNF receptor-stimulating activity of the two TNF forms. While transmembrane TNF activates TNFR1 and TNFR2 signaling with high efficacy, binding of soluble TNF results only in the case of TNFR1 in strong and general receptor activation (Wajant et al., 2003; Figure 1). TNFR1 is expressed by almost any cell type. TNFR2 expression, however, is rather restricted to certain cell types, including myeloid cells, regulatory T-cells, glial cells and some endothelial cell types, but can also be induced in epithelial cells, fibroblasts and certain T- and B-cell subsets (Medler and Wajant, 2019). TNFR2 is furthermore frequently expressed on hematopoietic malignancies and some solid tumors. TNF is not constitutively expressed and is instead readily induced in activated immune cells but it is also expressed by fibroblasts and endothelial and epithelia cells in response to proinflammatory triggers and cytokines including TNF itself (Pauli, 1994;Medler and Wajant, 2019).

## TNFR1-Related Signaling Pathways

**FIGURE 2:**
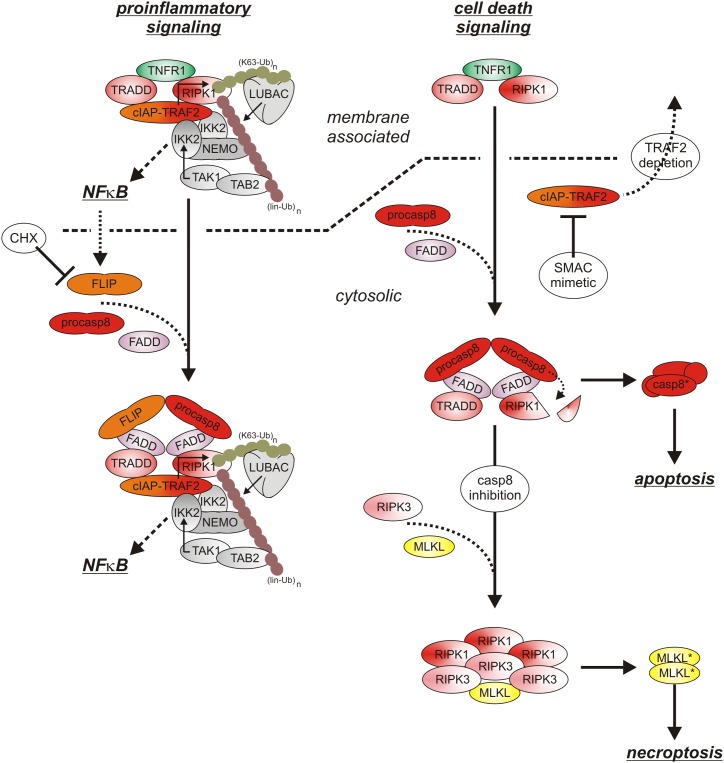
TNFR1 signaling. The default state of TNFR1 signaling results in activation of proinflammatory pathways such as the classical NFκB pathway. If FLIP proteins and the TRAF2-cIAP1/2 complexes are limited, e.g., due to CHX treatment and/or TRAF2-cIAP1/2 depletion), the quality of TNFR1 signaling shifts to apoptosis. Not before caspase-8 activity is limited under apoptotic conditions, there is finally necroptosis induction. For more details see text.

After binding of soluble or membrane-bound TNF, the DD-containing cytoplasmic proteins TNFR1-associated death domain (TRADD) and receptor interacting protein kinase-1 (RIPK1) recruit to TNFR1 due to DD–DD interactions (Figure 2). Deficiency or knock-down of RIPK1 enhance recruitment of TRADD and TRAF2 to TNFR1 (Devin et al., 2001; Jin and El-Deiry, 2006; Zheng et al., 2006; Fullsack et al., 2019). Deficiency or knock-down of TRADD, in contrast, consistently reduced TRAF2 recruitment in various studies and showed varying effects on RIPK1 recruitment (Jin and El-Deiry, 2006; Zheng et al., 2006; Ermolaeva et al., 2008; Pobezinskaya et al., 2008; Fullsack et al., 2019). Since TRAF2 interacts furthermore with high affinity with TRADD outside its DD (Park et al., 2000), these findings suggest that TNFR1-bound TRADD, and to a lesser extent TNFR1-bound RIPK1, recruit TRAF2 homotrimers (or TRAF1-TRAF2 heterotrimers) into the TNFR1 signaling complex. With TRAF2 the E3 ligases cIAP1 and cIAP2, which already form complexes with TRAF2 homotrimers (or TRAF1-TRAF2 heterotrimers) in the cytoplasm, become co-recruited to the TNFR1 signaling complex (Wajant and Scheurich, 2011). The cIAPs modify various components of the TNFR1 signaling complex, in particular RIPK1, with K63-linked ubiquitin chains and create so binding sites for the E3 ligase linear ubiquitin chain assembly complex (LUBAC). The LUBAC then further modifies RIPK1 with linearly linked ubiquitin chains which allow the recruitment of the MAP3K transforming growth factor-β (TGFβ)–activated kinase-1 (TAK1) via the adapter protein TAK1-binding protein-2 (TAB2) and of the inhibitor of kappa B kinases (IKK) complex (Wajant and Scheurich, 2011; Brenner et al., 2015). TAK1 can now phosphorylate and activate the IKK2 subunit of the IKK complex and triggers this way the events of the classical NFκB pathway including phosphorylation and degradation of inhibitor of kappa B-alpha (IκBα), release and nuclear translocation of previously IkBα-inhibited NFκB dimers and transcription of various NFκB-regulated targets. The latter include IκBα itself but also other factors that in feedback loops can modulate TNFR1 signaling, e.g., the FADD-like ICE-inhibitory proteins (FLIPs), cIAP2, A20 and TRAF1 (Wajant et al., 2003). The described chain of events emerge from the plasma membrane located TNFR1 signaling complex (also named complex I) within seconds to very few minutes and allows production of functional relevant amounts of NFκB-regulated proteins in less than 1 h (Wajant and Scheurich, 2011; Brenner et al., 2015).

With time the TNFR1 signaling complex starts to internalize and this comes along with the release of the TNFR1-bound signaling molecules (Fritsch et al., 2017). In the cytoplasm the latter can form receptor-free cytoplasmic complexes (also named complex IIa and IIb) with FADD, caspase-8 and RIPK3 which context-dependent enable induction of apoptosis or necroptosis (Brenner et al., 2015). Since TRADD, RIPK1, TRAF2 and the cIAPs are also part of the initially formed TNFR1-associated signaling complex, it is tempting to speculate that these molecules as a whole can dissociate from TNFR1 to recruit then FADD/caspase-8 and to act as a “condensation nucleus” to recruit RIPK3 and additional RIPK1 and RIPK3 molecules (Li et al., 2012). Noteworthy, there is evidence that TNFR1-induced activation of p38 and its downstream target MAPK kinase-activated kinase-2 (MK2) results in MK2-mediated phosphorylation of RIPK1 on serine 320 (human)/321(mouse) of TNFR1-associated RIPK1 but also of “free” cytosolic RIPK1 which hinders RIPK1 from acting as “condensation nucleus” of cytosolic complexes containing kinase active RIPK1 (Li et al., 2012; Jaco et al., 2017). Complex IIa-associated maturation of procaspase-8 dimers results in the release of mature heterotetrameric caspase-8 molecules composed of the two p18 and p10 subunits of a procaspase-8 dimer. Subsequent apoptosis induction is typically suppressed by constitutive and NFκB-induced expression of FLIP proteins and K63-ubiquitination of RIPK1 (Brenner et al., 2015). Thus, cells are normally resistant against TNF-induced apoptosis as long as FLIP expression/induction is not inhibited (e.g., by CHX or IKK inhibitors) and/or RIPK1 K63 ubiquitination is not prevented (e.g., by SMAC mimetics or TRAF2 depletion) (Brenner et al., 2015; Annibaldi and Meier, 2018). Noteworthy, complex IIa-associated active caspase-8 and caspase-8/FLIP_L_ heterodimers, which have a limited enzymatic activity, cleave RIPK1 and RIPK3 and prevent so complex IIb triggered formation of necroptosis-inducing oligomeric RIPK1/RIPK3 aggregates (Brenner et al., 2015). Moreover, K63- and linear ubiquitination of RIPK1 and activation of TAK1 and IKK not only stimulate the anti-apoptotic classical NFκB pathway but also inhibits cytotoxic RIPK1 activation by phosphorylation in its intermediate domain (S320 of human RIPK1, S321 of murine RIPK1) directly (IKK and TAK1) and indirectly (TAK1) via activation of the p38-MK2 dyad (Dondelinger et al., 2015, 2017; Geng et al., 2017; Jaco et al., 2017; Menon et al., 2017). TNF-induced necroptosis therefore only occurs when caspase-8 activation fails in cells with a compromised TNFR1-TRAF2-cIAP1/2-LUBAC-TAK1-IKK sequence (Figure 2). Apoptotic cells release membrane-enclosed apoptotic vesicles containing the cellular content of the dying cell which are cleared by macrophages without triggering inflammation. In contrast, necroptosis is a lytic form of cell death releasing intracellular DAMPs and proinflammatory cytokines and thus promotes inflammation (Kearney and Martin, 2017; Frank and Vince, 2019). Since ongoing TNFR1 signaling is *per se* highly inflammatory, TNF-induced necroptosis might nevertheless dampen inflammatory TNF effects under certain circumstances (Kearney and Martin, 2017). Thus, the inflammatory net effect of TNFR1 *in vivo* is determined by the complex interplay of TNFR1-induced classical NFκB signaling, apoptosis and necroptosis.

In context of TNFR1 signaling, TRADD, RIPK1, TRAF2 and TAK1 are not only of central relevance for the activation of the classical NFκB pathway and suppression of the cell death inducing capacity of TNFR1 but are also responsible for triggering the MAP kinase cascades leading to the activation of JNK and p38 (Wajant et al., 2003). Since the relevance of TNFR1-induced activation of JNK and p38 signaling have been poorly addressed so far in macrophages, it will not been addressed further in this review. The same applies for TNFR1-induced DD-independent activation of the neutral sphingomyelinase and ERK signaling pathways.

## TNFR2-Related Signaling Pathways

**FIGURE 3:**
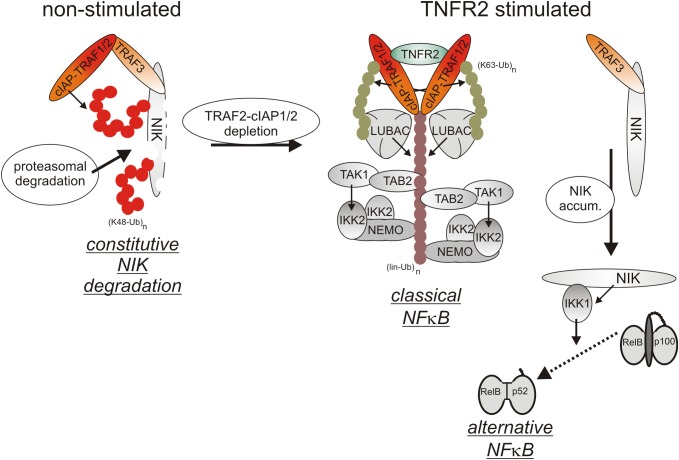
TNFR2 signaling. TNFR2 efficiently recruits TRAF2-cIAP1/2 and TRAF1-TRAF2-cIAP1/2 complexes which allow activation of the classical NFκB pathway. TNFR2 recruitment of TRAF2-cIAP1/2 and TRAF1-TRAF2-cIAP1/2 complexes concomitantly depletes the cytosolic pool of these proteins and hinder them so to trigger degradation of the alternative NFκB pathway stimulating kinase NIK. Thus, TNFR2 also activates the alternative NFκB pathway.

Initially, oligomerized TNFR2 recruit TRAF2 along with its tightly associated binding partners TRAF1, cIAP1 and cIAP2 what resembles the indirect, TRADD/RIPK1-mediated recruitment of these proteins in context of TNFR1 signaling. Although there is no evidence for a role of TRADD and/or RIPK1 in TNR2 signaling, the LUBAC as well as the IKK complex are also recruited to the TNFR2 signaling complex (Figure 3), but less efficient as in the case of TNFR1 (Wicovsky et al., 2009; Borghi et al., 2018). TNFR2 stimulation results therefore in activation of the classical NFκB pathway, too.

Cell surface expression levels of TNFR2 reach often > 10000 molecules per cell (e.g., Gehr et al., 1992; Medvedev et al., 1996) and are regularly much higher than those of TNFR1 which are typically in the range of a few hundred to 1-3 thousand molecules per cell (e.g., Thoma et al., 1990; Gehr et al., 1992). Recruitment of TRAF2-cIAP1/2 and TRAF1-TRAF2-cIAP1/2 complexes to TNFR2 can therefore lead to a significant depletion of these complexes in the cytoplasm and may thus affect other activities of these molecules (Duckett and Thompson, 1997; Fotin-Mleczek et al., 2002; Li X. et al., 2002). Indeed, TRAF2 and the cIAPs are constitutively engaged in the cytoplasm in the inhibition of the alternative/non-canonical NFκB pathway which is of special relevance for the control of the activity of p52-RelB NFκB dimers (Sun, 2017). By virtue of its ability to reduce the cytosolic pool of TRAF2-containing complexes, TNFR2 is thus able to activate the alternative NFκB pathway (Rauert et al., 2010). In detail, TRAF2 recruits its binding partners cIAP1/2 to TRAF3 and the TRAF3 interacting MAP3-kinase NIK. The cIAPs ubiquitinate NIK with K48-linked ubiquitin chains and thereby promote the proteasomal degradation of this constitutively active kinase. TNFR2-induced depletion of cytosolic TRAF2-cIAP1/2 complexes results therefore in the accumulation of active NIK and NIK-mediated phosphorylation of the NFκB precursor protein p100. Phosphorylated p100 becomes K48-ubiquitinated and is then processed by the proteasome to the NFκB transcription factor subunit p52, thus resulting in the activation of p100-containing NFκB dimers (Sun, 2017). Although it has been found that depletion of TRAF2-cIAP1/2 and TRAF1-TRAF2-cIAP1/2 complexes is fully sufficient to interfere with the cytoplasmic activities of these complexes (Fotin-Mleczek et al., 2002), the depletion effect might be enhanced and sustained further by TNFR2-triggered proteasomal degradation of TRAF2 and cIAP2 (Duckett and Thompson, 1997; Li X. et al., 2002). TNFR2-induced depletion of the cytosolic pool of TRAF2-cIAP1/2 and TRAF1-TRAF2-cIAP1/2 complexes can also limit the availability of these proteins for other receptors. Indeed, it has been observed that prestimulation of TNFR2 (or similarly acting TNFRSF receptors such as Fn14) affects recruitment of TRAF2 and cIAPs to TNFR1 and thereby attenuates the ability of TNFR1 to stimulate classical NFκB signaling (Fotin-Mleczek et al., 2002). Due to the relevance of TRAF2 and cIAPs for preventing apoptosis and necroptosis in context of TNFR1 signaling, TNFR2-mediated depletion/degradation of these molecules can result in enhanced TNFR1-induced cell death in macrophages as is discussed below in detail.

In a cell type-specific manner TNFR2 can also activate the tyrosine kinase bone marrow kinase on chromosome X (BMX) and the phosphatidylinositol 3-kinase (PI3K)/protein kinase B(Akt) pathway (Pan et al., 2002; So and Croft, 2013). The latter presumably occurs TRAF2-dependent because TRAF2 has been implicated in Akt activation by various receptors including TNFR1 and the TNFR2-related TNFRSF receptor CD40 (Davies et al., 2005; Zhu et al., 2016). In contrast, TNFR2-induced BMX activation occurs independently from TRAF2 (Pan et al., 2002). The molecular mechanisms used by TNFR2 to activate BMX and PI3K/Akt are, however, poorly investigated and their relevance in macrophages has not been addressed so far.

In sum, TNFR1 and TNFR2 are differently activated by soluble and membrane TNF, induce production of their own ligand in some cells, engage receptor-specific but also common pathways and the various TNFR1/2-associated signaling pathways are, last but not least, interconnected by regulatory circuits. TNF and its receptors therefore constitute a complex signaling network what is reflected by the fact that additive, synergistic or even antagonistic effects have been reported for the two TNF receptor types.

## The TNF-Induced Cytotoxic Signaling Network in Macrophages

The complexity of the TNF-TNFR1-TNFR2 signaling system is especially relevant in macrophages because this cell type not only co-expresses TNFR1 and TNFR2 but also produces high amounts of TNF upon stimulation of a variety of receptors including all types of PRRs and various members of the TNFRSF. Of central relevance for the upregulation of TNF is the activation of the classical NFκB pathway. Macrophage-produced TNF not only mediates the proinflammatory and cytotoxic activities of this cell type but also regulates in an autocrine fashion the viability and activation status of macrophages. Indeed, there are a variety of examples of pathogen-induced macrophage cell death that crucially involves TNF (Table 1). An intensively studied example is killing of human alveolar and monocyte-derived macrophages by *in vitro* infection with mycobacteria (Keane et al., 1997; Balcewicz-Sablinska et al., 1998, 1999; Bermudez et al., 1999). Interestingly, mycobacteria infection also results in macrophage production of IL-10 which in turn triggers TNFR2 shedding resulting in TNF neutralization by soluble TNFR2 and reduced apoptosis (Balcewicz-Sablinska et al., 1998, 1999). TNF-induced cell death in mycobacteria-infected murine macrophages and macrophage cell lines has been attributed to TNFR1-induced caspase-8 activation and concomitant TNFR1-induced reactive oxygen species (ROS)-mediated activation of apoptosis signaling kinase-1 (ASK1) which promotes c-Cbl-mediated ubiquitination and degradation of the short FLIP isoform (FLIP_S_) (Bhattacharyya et al., 2003; Kundu et al., 2009). Worth mentioning *Mycobacterium tuberculosis* can also trigger RIPK3/MLKL-mediated cell death by tuberculosis necrotizing toxin-mediated NAD^+^ depletion independently from TNF and RIPK1 signaling (Pajuelo et al., 2018).

**Table 1:** Pathogen-induced TNF-mediated macrophage killing.

Pathogen	TNF-inducing factor/ mechanisms	Evidence	References
*Mycobacterium avium*	–	Anti-TNF	Balcewicz-Sablinska et al., 1999; Bermudez et al., 1999
*Mycobacterium tuberculosis*	–	Anti-TNF	Balcewicz-Sablinska et al., 1998; Rojas et al., 1999
*Mycobacterium tuberculosis*	Mce4	Anti-TNF	Saini et al., 2016
*Mycobacterium tuberculosis*	PGRS33 > TLR2	Anti-TNF	Basu et al., 2007
*Helicobacter pylori*	JHP0290	Anti-TNF	Pathak et al., 2013
*Salmonella enterica*	OMP96-induced cell stress	Anti-TNF	Chanana et al., 2006, 2007
**Ureaplasma urealyticum**	–	Anti-TNF	Li Y.H. et al., 2002
*–*	LPS	Anti-TNF	Xaus et al., 2000
*Bacillus Calmette-Guérin*	–	TNFR1 KO	Rodrigues et al., 2013
*Yersinia pseudotuberculosis*	–	TNFR1 KO	Peterson et al., 2016


## TNFR1-Dependent Necroptosis in Macrophages

Studies with SMAC mimetics (IAP antagonists) depleting cIAP1/2 and the cIAP1/2-related XIAP molecule as well as evaluation of XIAP and cIAP1/2 knockout cells revealed that these molecules are crucial for the survival of primary murine bone marrow-derived macrophages (McComb et al., 2012; Wong et al., 2014). The survival function of XIAP, cIAP1 and cIAP2 in this scenario could be traced back to the suppression of TNF-induced necroptosis (McComb et al., 2012; Wong et al., 2014). Studies with murine macrophages genetically deficient for TNFR1, TNFR2 and TNF together with the use of TNFR1- and TNFR2-specific agonists and antagonists revealed furthermore that both TNF receptors cooperate in triggering necroptotic cell death (Legarda et al., 2016; Siegmund et al., 2016; Lawlor et al., 2017). While exogenous TNF and/or autocrine TNF produced in response to TNFR1 and TNFR2 activation deliver a potential trigger for TNFR1-induced necroptosis, TNFR2 signaling enables realization of the necroptotic potential of activated TNFR1 by depletion of the cytosolic pool of TRAF2-cIAP1/2 complexes (Ruspi et al., 2014; Siegmund et al., 2016). As already mentioned above, some pathogens, e.g., mycobacteria, trigger IL-10-mediated shedding of TNFR2 to dampen/escape autocrine TNF killing (Balcewicz-Sablinska et al., 1998, 1999). The protective effect of TNFR2 shedding has so far mainly be attributed to the neutralization of TNF by the soluble TNFR2 ectodomain (Balcewicz-Sablinska et al., 1998, 1999). In light of the pro-necroptotic activity of TNFR2 in macrophages identified in recent years, however, it appears plausible that the protective effect of TNFR2 shedding is also due to the inhibition of the pro-cell death activities of TNFR2. RIPK1 and RIPK3 trigger execution of necroptotic cell death mainly by activation of mixed lineage kinase domain-like (MLKL) protein which forms cell-lytic plasma membrane pores and stimulation of mitochondrial production of reactive oxygen species (ROS) (Roca and Ramakrishnan, 2013; Fulda, 2016). Since ROS can promote TNF mRNA expression in human and murine macrophages (Gossart et al., 1996; Chandel et al., 2000; Kono et al., 2000; Brown et al., 2004), this arm of the necroptotic response might close a feed forward loop in TNF-induced necroptosis.

In accordance with the established anti-necroptotic activity of caspase-8, robust/maximal necroptosis induction by TNF in macrophages requires caspase-8 inhibition (McComb et al., 2012; Siegmund et al., 2016). In most studies, the latter has been achieved artificially by the use of caspase-8 inhibitors or genetic deletion of the caspase-8 gene. This raises the question for the physiological/pathophysiological conditions under which necroptosis occurs. A first pathophysiological scenario where caspase-8 is limited are tumor cells that evade from apoptotic surveillance mechanisms by down-regulation of caspase-8 expression, for example by DNA methylation or caspase-8 gene deletion (e.g., references Grotzer et al., 2000; Teitz et al., 2000; Fulda et al., 2001; Shivapurkar et al., 2002; Hopkins-Donaldson et al., 2003). Necroptosis induction in caspase-8 low tumor types, however, has been poorly investigated so far. A second scenario where caspase-8 activity is limited and necroptosis gain relevance is in cells infected by viruses encoding caspase-8 inhibitory proteins. Several viral inhibitors of caspase-8 have been identified in recent years and include CrmA from cowpox virus and the baculovirus-encoded p35 protein (Ray et al., 1992; Zhou et al., 1997; Xu et al., 2001) but also proteins of clinically important human viruses. The human cytomegalovirus (CMV) gene UL36 encodes the viral inhibitor of caspase-8-induced apoptosis (vICA) protein which is conserved in primates and rodents and the ribonucleotide reductase R1 subunits of herpes simplex virus types 1 and 2 also act as caspase-8 inhibitors (McCormick, 2008; Dufour et al., 2011). Please be aware that some of these viral proteins not only inhibit caspase-8 but also other caspases including caspase-1 which is of crucial relevance for signaling by inflammasomes. The relevance of caspase-8 inhibition and sensitization for necroptosis for the *in vivo* effects of these pathogenic factors can therefore be difficult to define.

## TNF-Induced Necroptosis and Its Integration in PRR Signaling

In accordance with the fact that PRRs strongly stimulate TNF production by macrophages, it has been described that induction of TNF and TNFR2-mediated TRAF2 depletion contribute to macrophage necroptosis induced by certain TLRs, including TLR4 and TLR3 (Kaiser et al., 2013; Legarda et al., 2016; Siegmund et al., 2016; Lawlor et al., 2017). TNF-independent necroptosis induction by TLR4 and by TLR3, however, has also been described (He et al., 2011; McComb et al., 2014). The varying relevance of autocrine TNF production for TLR4-induced necroptosis presumably reflects the use of different doses of the TLR4 agonist lipopolysaccharide (LPS) in the cited studies. Indeed, it has been described that high doses of LPS directly activate necroptotic signaling so that the effect/contribution of autocrine TNF-induced necroptosis is masked (Legarda et al., 2016). Studies with XIAP-deficient bone marrow progenitor cell-derived dendritic cells revealed an unexpected inhibitory role of XIAP on TNF signaling (Yabal et al., 2014) which was later on confirmed in murine bone marrow-derived macrophages (Lawlor et al., 2015, 2017). It turned out that LPS-stimulated XIAP-deficient macrophages, despite unchanged TNF production, elicit an enhanced cell death response, inflammasome activation and IL-1β secretion in a TNF-dependent manner (Yabal et al., 2014). Worth mentioning XIAP-deficiency showed no effect on TNF-induced activation of the classical NFκB pathway, p38 MAPK activation and TNFR1 signaling complex formation (Yabal et al., 2014). Thus, XIAP might preferentially affect TNF-related activities in macrophages that require RIPK3. Indeed, TNF-induced IL-1β secretion, caspase-8 activation and cell death induction were blocked in dendritic cells derived of XIAP/RIPK3 DKO mice (Yabal et al., 2014).

**FIGURE 4:**
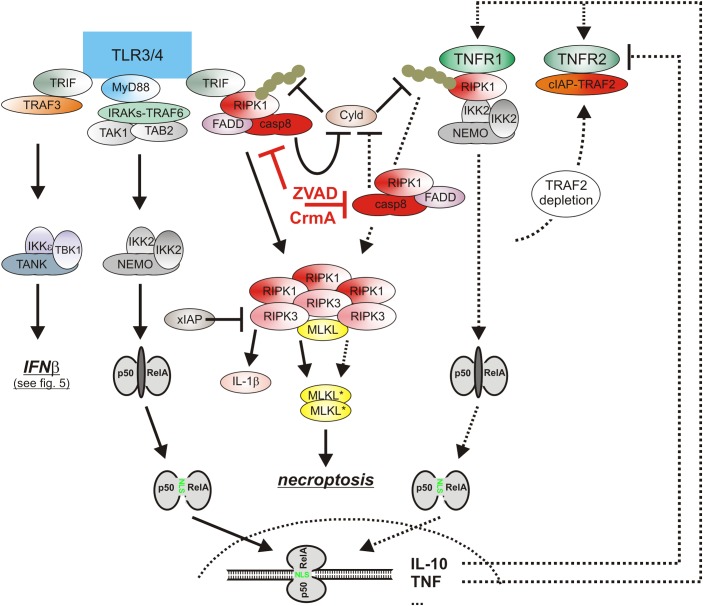
The cytotoxic TLR-TNF signaling network. TNF expression is strongly upregulated by PRRs such as TLR3 and TLR4 via the classical NFκB pathway. TNF via the two TNF receptors can then cooperate with TLR3/4-TRIF-RIPK1 axis to trigger apoptosis or necroptosis. For details see text. Please note, necroptosis only takes place when caspase-8 activity is limited, e.g., by viral caspase-8 inhibitors.

Lipopolysaccharide-induced TLR4 signaling has not only the potential to trigger necroptosis via induction of endogenous TNF but also adjusts the necroptotic sensitivity of macrophages for TNF in a complex manner (Figure 4). On the one side, TLR4 induces type I interferons which have various pro-necroptotic effects as is discussed below in detail. One the other side, TLR4 activates caspase-8 in a TNF-independent manner and promotes so the “inactivating” cleavage of the deubiquitinase Cyld (Legarda et al., 2016; Figure 4). Cyld removes K63-linked polyubiquitin chains from RIPK1 and interferes so in context of TNFR1 signaling with the recruitment and survival functions of the TAB2-TAK1 and IKK complexes. The LPS-induced caspase-8-mediated degradation of Cyld thus desensitizes macrophages for necroptosis. TLR4-induced caspase-8 activation requires the TLR4-RIPK1 linking adapter protein Toll/interleukin-1 receptor domain-containing adaptor protein inducing interferon (TRIF) and is dependent on RIPK1 and FADD (Weng et al., 2014; Legarda et al., 2016; Peterson et al., 2016). Caspase-8 activation by TLR4 thereby closely resembles the mechanisms of TNFR1-induced TRADD-RIPK1-mediated stimulation of the FADD-caspase-8 dyad.

Studies with human-induced pluripotent stem cells (hiPSCs) showed that differentiated RIPK1-deficient macrophages progressively undergo autocrine TNF-dependent cell death (Buchrieser et al., 2018). Thus, RIPK1 can also elicit anti-necroptotic activity in context of TNF signaling in macrophages. However, the cues defining the net quality of RIPK1 effects in macrophages are elusive. Noteworthy, addition of exogenous soluble TNF not further enhanced endogenous-TNF dependent cell death of RIPK1 KO hiPSCs while LPS- and poly(I:C)-induced TNF-independent necroptosis was enhanced (Buchrieser et al., 2018). Since soluble TNF poorly stimulate TNFR2, it is tempting to speculate that TNFR2-mediated cell death sensitization is the limiting step in TNF-induced cell death in this model.

## Cooperation of TNF and Type I Interferons in Necroptotic Signaling in Macrophages

**FIGURE 5:**
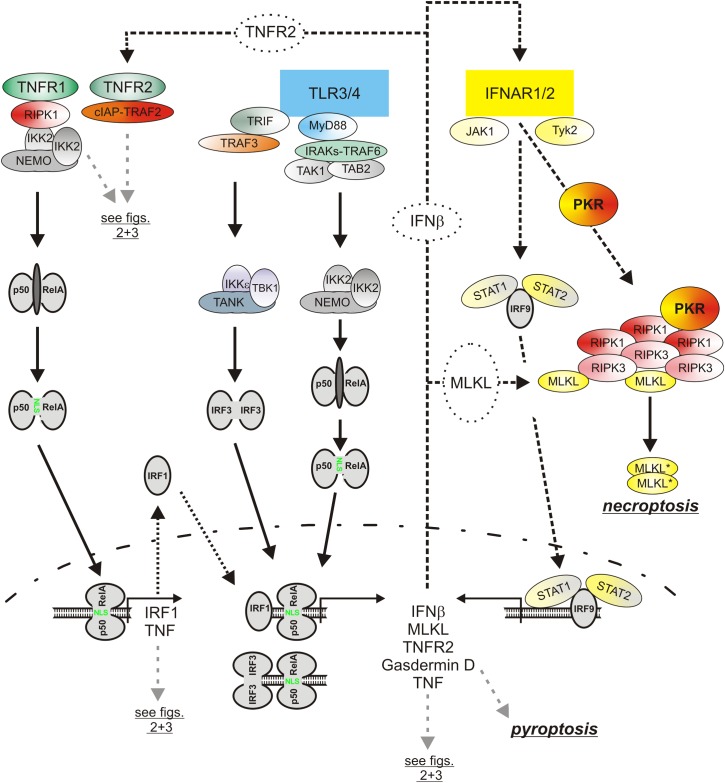
The role of type I interferons in the cytotoxic TLR-TNF signaling network. TNF via the classical NFκB pathway induces IRF1 which in turn stimulates in cooperation with the classical NFκB pathway the expression of low amounts of IFNβ. The latter boosts its own expression by autocrine signaling but also the expression of pro-necroptotic proteins, such as TNFR2 and MLKL. PRRs, e.g., TLR4, often co-induce TNF and IFNβ expression. Thus, cytotoxic signaling by PRRs, IFNβ, and TNF are interconnected in various ways in macrophages by feed forward loops. Please be aware, for simplicity functional relevant phosphorylation and ubiquitination events are not included. For more details see text.

Besides the classical NFκB pathway, the type I interferon pathway is a second major signaling pathway which is regularly engaged by PRRs (Figure 5). The central elements of the type I interferon pathway are the kinases TANK-binding kinase 1 (TBK1) and inhibitor of kappaB kinase ε (IKKε) which phosphorylate and activate the interferon-β gene inducing transcription factor interferon regulatory factor 3 (IRF3). In context of TLR3 and TLR4 signaling the adapter proteins TRIF and TRAF3 mediate activation of TBK1/IKKε and IRF3. Together with the in parallel activated classical NFκB pathway, the type I interferon pathway stimulates the production of the type I interferons (Ikushima et al., 2013). Thus, TNF and type I interferons are co-produced by pathogen challenged macrophages. Similarly to TNF, type I interferons not only act as effector molecules of activated macrophages but also retroact on the macrophages. Moreover, there is growing evidence that TNF and type I interferons cooperated in the control of macrophage viability (Figure 4). So it has been found that TNF/ZVAD- and LPS/ZVAD-induced necroptosis are blocked in macrophages derived of Interferon-α/β receptor alpha chain (IFNAR1) knockout mice (McComb et al., 2014; Legarda et al., 2016). The compromised necroptosis sensitivity correlated with reduced expression of the “pro-necroptotic” proteins TNFR2 and MLKL (Legarda et al., 2016). Reconstitution experiments revealed, however, that reexpression of TNFR2 and MLKL alone is not sufficient to restore necroptotic sensitivity for LPS pointing to additional type I interferon targets which are of relevance in necroptotic signaling. Indeed, there is evidence that TRIF-dependent induced type I interferons also promote the expression of Z-DNA binding protein-1 (Zbp1) and gasdermin D (GSDMD), which after cleavage can also cause lytic cell death (see below), via KAT2B- and p300-mediated histone 3 acetylation at lysine 27 (Li et al., 2018). Moreover, it has been shown that interferon-β activates protein kinase R (PKR) and promotes so its interaction with RIPK1 to trigger RIPK1/RIPK3-mediated necroptotic cell death (Thapa et al., 2013). The relevance of this mechanism for TNF-induced IFNAR1-dependent necroptosis in murine macrophages is, however, unclear. Last but not least, it has been found in caspase-8-inhibited macrophages that LPS initially induces TRIF-mediated type I interferon production which then trigger via the interferon-stimulated gene factor-3 (ISGF3) complex sustained RIPK3 phosphorylation and necroptosis by a yet poorly understood mechanism independent from PKR (McComb et al., 2014; Saleh et al., 2017).

Noteworthy, there is not only evidence that type I interferons contribute to TNF-induced necroptosis but vice versa also that TNF- and/or necroptosis-associated signaling contribute to the induction of type I interferons. TNF induces in macrophages not only NFκB-regulated genes but also, with delay, typical signal transducer and activator of transcription-1 (STAT1)- and interferon response proteins such as MX1, IRF7 and STAT1 itself (Yarilina et al., 2008). The stimulation of the transcription of the latter proteins is due to an indirect mechanism involving NFκB-mediated upregulation of IRF1 via both TNF receptors and subsequent induction of low concentrations of IFNβ by the joint action of IRF1 and NFκB transcriptions factors (Yarilina et al., 2008). TNF and TNF-induced IFNβ cooperate then in the sustained and strong expression of NFκB/STAT co-regulated inflammatory factors such as CCL5, CXCL10 and CXCL11 and also maintain IFNβ expression (Yarilina et al., 2008). Whether TNF-induced IFNβ production is of relevance for TLR4-triggered necroptosis appears, however, unlikely as the TLR4-TRIF-IRF3 axis is already sufficient to mount a strong type I interferon response. There is, however, evidence from studies with LPS/ZVAD-treated macrophages that TBK1, IKKε, RIPK1 and RIPK3 form a high molecular weight complex which via RIPK3 promote TBK1/IKKε signaling (Saleh et al., 2017). Thus, TNF-induced RIPK1/RIPK3 activation in course of necroptotic signaling might have the potential to boost IFNβ production but this has not been evaluated yet.

## TNF and Caspase-8-Mediated Inflammasome Activation and Pyroptosis

Besides apoptosis and necroptosis, pyroptosis is a third form of programmed cell death which is of particular relevance in macrophages (Man et al., 2017). Pyroptosis is a strongly proinflammatory form of lytic cell death which is triggered downstream of inflammasome complexes by caspase-1 mediated cleavage of gasdermin D (GSDMD). The N-terminal p30 cleavage product of GSDMD forms then large pores in the plasma membrane and executes so cell lysis (Man et al., 2017). Inflammasome-triggered pyroptosis of macrophages and intestinal epithelial cells is of special relevance for combating infection by intracellular bacteria (Frank and Vince, 2019). Recent studies showed that Yersinia bacteria by help of their TAK1 inhibitory acetyltransferease YopJ triggers RIPK1-mediated activation of caspase-8 (Orning et al., 2018; Sarhan et al., 2018). Interestingly, this not only results in effector caspase activation and apoptosis but also in caspase-8-mediated, thus non-canonical GSDMD cleavage, GSDMD-mediated NLRP3 inflammasome activation and pyroptosis (Orning et al., 2018; Sarhan et al., 2018). In one of these reports, it has been furthermore shown that cell death induction and IL-1β production by a mixture of pharmacological TAK1 inhibitors and TNF are reduced in GSDMD-deficient murine macrophages (Orning et al., 2018). Moreover, Yersinia-induced cell death was reduced in murine macrophages deficient for TLR4, TRIF or TNFR1 (Orning et al., 2018). Thus, under appropriate conditions TNF may also trigger GSDMD-dependent non-canonical inflammasome activation and pyroptosis, too (Figure 5). GSDMD is presumably directly cleaved by caspase-8 in these scenarios because TAK1 inhibition/LPS-induced generation of the pore forming p30 GSDMD fragment occurred in caspase-3/-7 double-deficient macrophages and GSDMD coimmunoprecipitated furthermore with caspase-8 (Orning et al., 2018; Sarhan et al., 2018). The mechanisms described are presumably of broader relevance as various other pathogenic bacteria and viruses, e.g., enteroviruses, pseudomonas and vibrio also utilize TAK1 inhibitory proteins (Zhou et al., 2013; Lei et al., 2014; He et al., 2017; Rui et al., 2017). RIPK1-mediated caspase-8 activation is inhibited by cIAPs (see above). Since Yersinia, LPS and TNF trigger cIAP depletion via the TLR4-TRIF pathway and the TNFR2-TRAF2 axis, it appears possible that cIAP depletion contributes to the pyroptotic RIPK1-caspase-8-GSDMD signaling branch but this issue has not been experimentally addressed yet.

Most pathogens activate in macrophages several types of inflammasome complexes. *Yersinia pestis* for example not only activates the NLRP3 inflammasome but also the pyrin inflammasome (Philip and Brodsky, 2012; Jamilloux et al., 2018). The sensor protein pyrin detects Rho GTPases molecule species which are inhibited by bacterial toxins and forms then an inflammasome with ASC and procaspase-1 (Jamilloux et al., 2018). TNF and various other PPR-induced cytokines, including type I interferons, stimulate the expression of pyrin in macrophages (Centola et al., 2000). Thus, the TNF triggered pyroptotic RIPK1-caspase-8-GSDMD signaling axis might further cooperate with TNF/interferon-induced pyrin expression and enhanced pyrin inflammasome activity to promote macrophage pyroptosis (Figure 5). Indeed, a contribution of TNF-induced pyrin expression to pyrin inflammasome activation, IL-1β production and pyroptosis induction has been recently demonstrated for clostridium difficile toxin B (Sharma et al., 2019).

Just recently two studies demonstrated that intrinsic, thus mitochondria-dependent apoptosis in bone marrow-derived macrophages is accompanied by activation of the NLPR3 inflammasome and IL-1β activation (Chauhan et al., 2018; Vince et al., 2018). Noteworthy, the latter was not only due to activation of the NLRP3 inflammasome but has also been traced back to inflammasome-independent IL-1β processing by caspase-8. In context of intrinsic apoptosis caspase-8 is directly activated by processing by effector caspases and indirectly by cIAP1/2 depletion and subsequent RIPK1 kinase activation. Caspase-8-activation and cIAP1/2 depletion can also be triggered by TNFR1 (or other death receptors) and TNFR2. It is thus well conceivable that TNF triggers this unusual proinflammatory mode of apoptosis, especially under circumstances where TNFR1-induced caspase-8 is insufficiently blocked. Future studies have to show whether the proinflammatory activities of caspase-8 (GSDMD cleavage, IL-1β processing) gain relevance for the biology and pathophysiology of TNF *in vivo*. Noteworthy, the pyroptotic GSDMD p30 fragment is able to trigger mitochondrial ROS production (Platnich et al., 2018) and the ROS in turn are established inducers of the NLRP3 inflammasome (Tschopp and Schroder, 2010) and, as mentioned before, of TNF expression (Gossart et al., 1996; Chandel et al., 2000; Kono et al., 2000; Brown et al., 2004). Thus, the GSDMD p30-ROS axis might auto-amplify p30 production by two feed forward loops, first by NLRP3 inflammasome activation and second by TNF-induced caspase-8 activation. TNF itself is furthermore able to trigger mitochondrial production of ROS in macrophages and thus might further enhance these feed forward loops.

## Conclusion and Perspective

There are a considerable number of high quality publications addressing the role of TNF in the life death balance of macrophages. In sum, these studies show that the effect of TNF on macrophage viability not only depends on the integrated and complex activity of the TNFR1-TNFR2 signaling network but also from its crosstalk with other, equally complex signaling systems engaged by PRR-, inflammasomes and interferons. It is thus not really surprising that the precise net-effects of TNF on macrophages in infection diseases and cancer are still poorly predictable. Indeed, it is not even clear whether and if yes to which extend, the reported effects of TNF on macrophages are generalizable to all types of macrophages. Likewise, it is unclear under which *in vivo* conditions which of the various individual TNF-related signaling mechanisms gain dominance. Thus, future studies must show whether there are key factors that determine the quality of TNF signaling on macrophage viability. It appears particularly important to learn more about the crosstalk of concomitantly occurring signaling paths engaged by TNF and other inducers of macrophage cell death. Last but not least, it will be important for the understanding of the role of TNF for macrophage biology to learn more about the systemic immunological net-effects triggered by timely limited “immunogenic” death versus persistent inflammatory activation of macrophages.

## Author Contributions

All authors listed have made a substantial, direct and intellectual contribution to the work, and approved it for publication.

## Conflict of Interest Statement

The authors declare that the research was conducted in the absence of any commercial or financial relationships that could be construed as a potential conflict of interest.
